# Adoptive Cell Therapies for Glioblastoma: A Quest for Cures from Within

**DOI:** 10.3390/biology15080614

**Published:** 2026-04-13

**Authors:** Jia-Shiun Leu, Xin Ge, Charles Robin Yu, Guang Peng, Jiyong Liang

**Affiliations:** 1 Institute of Molecular Medicine, McGovern Medical School, University of Texas Health Science Center at Houston, Houston, TX 77030, USA; 2 Department of Experimental Therapeutics, The University of Texas MD Anderson Cancer Center, Houston, TX 77030, USA; cryu1@mdanderson.org; 3 Department of Neuro-Oncology, The University of Texas MD Anderson Cancer Center, Houston, TX 77030, USA

**Keywords:** adoptive cell therapy, glioblastoma, CAR-T, tumor-infiltrating lymphocyte, tumor microenvironment

## Abstract

Glioblastomas (GBMs) are the most aggressive form of brain cancer recalcitrant to both current standard-of-care and immune checkpoint therapies that benefit other cancer patients. Adoptive cell therapies (ACT) using patients’ own immune cells have long been explored as a treatment strategy, including the historically studied lymphokine-activated killer (LAK) cells, the evolving chimeric antigen receptor (CAR) directed immune cells, the newly emerging tumor-infiltrating T lymphocyte (TIL) therapies, and others. Preclinical and clinical studies have shown promise but also highlighted significant challenges. In this review, we summarize these findings, highlight recent developments, discuss current limitations, and emphasize how ACT may benefit from contemporary and future insights into the co-evolution of TILs with other cells within the GBM tumor microenvironment (TME).

## Simple Summary

Immunotherapy has led to major breakthroughs in recent years. More than 40 immunotherapy drugs are now approved by the U.S. Food and Drug Administration, treating over 30 types of human cancer. However, glioblastoma, a form of aggressive brain cancer, has not responded well to most common immunotherapy approaches including immune checkpoint blockade antibodies and adoptive cell therapies utilizing engineered patient T lymphocytes. Based on our experience and a review of laboratory and clinical studies, we found that limited persistence poses a significant challenge to the development of adoptive cell therapies. We propose strategies to improve how well adoptive immune cells adapt to the challenging conditions and stay functional within glioblastoma tumors, with the goal of achieving more durable and meaningful outcomes for patients.

## 1. Early Approaches

Adoptive cell therapy (ACT) for malignant glioma originated from early studies using lymphokine-activated killer (LAK) cells generated by culturing autologous peripheral blood lymphocytes obtained from brain-tumor patients with recombinant IL2 [1,2,3]. The standard approach involved collecting and stimulating patients’ peripheral lymphocytes with IL2 in vitro for a few days, after which the LAK cells hence generated were injected via the intracranial tumor (ICT) route directly into the brain tissue surrounding the cavity left by operative tumor debulking. Typically, a single or multiple doses of IL2 were infused to support the viability and function of the injected cells.

Initial reports in the late 1980s demonstrated that autologous LAK cells could be administered intracerebrally into patients with tumor-selective cytotoxicity [4,5]. Subsequent studies confirmed the safety and feasibility of the therapy [6,7], and some studies showed a favorable survival benefit [8,9,10]. However, attempts to enhance therapeutic efficacy through more aggressive post-transplant administration of IL2 did not improve outcomes and led to neurologic side effects in all patients, demonstrating limitations of the approach [11]. In parallel, large multicenter studies in other cancer types showed that LAK therapy offered limited and inconsistent clinical benefit, coupled with high toxicity [12,13,14,15]. As a result, clinical use of LAK cells for glioma treatment was eventually discontinued.

Notably, traditional LAK cell products were derived from peripheral leukocytes, which presumably comprise limited amounts of tumor-reactive immune effector cells. Additionally, the cells were produced without depleting monocytes and thus were not pure populations of lymphoid effectors. In addition to this heterogeneity, LAK cells are often not cancer specific. Retrospectively, the myeloid component of LAK cell products activated through either direct or indirect mechanisms may have contributed to inadvertent immune suppression. Furthermore, IL2 levels are minimal in the gliomas and are unrelated to TIL abundance, further arguing against the role of IL2 in T cell fitness within the glioma tumor microenvironment and the rationale for IL2-based LAK therapy in gliomas. Despite the cytotoxicity and limited clinical impact of IL2-based LAK therapy in gliomas, the discovery nevertheless highlighted the need for more tolerant and specific adoptive cell therapies.

## 2. CAR-T Cell Therapy

### 2.1. Overview

The focus of ACT for gliomas has shifted to chimeric antigen receptor (CAR)-T cells since the 2010s, emerging as one of the mainstream strategies of immunotherapy development. CAR-T cells were originally engineered to achieve both anti-tumor specificity and potency, as provided by the rational design of the chimeric structure comprising a high-affinity single-chain fragment variable (scFv) derived from a target-specific antibody fused to physiological intracellular signaling domains mediating T cell activation [16]. In addition to using scFv antigen-binding domains for target recognition, several unconventional approaches including physiological and peptide ligands of selective receptors have also been developed. The intracellular domains are commonly derived from CD28, CD3ζ chain, and 4-1BB (CD137), mimicking both T cell receptor (TCR) and co-stimulatory signaling to fulfill optimal T cell activation, expansion, function, and persistence [17,18,19,20]. Further, CAR structures can be fine-tuned to achieve more desirable target recognition and CAR-T cell function or to include inducible mechanisms to turn on or off CAR expression. Upon target recognition, CAR-T cells engage and fight cancer cells, leading to selective cytolytic or apoptotic cell death in cancer cells within a few hours. Clinical administration of autologous CAR-T cells targeting the B cell antigen CD19 has produced an unprecedented therapeutic outcome in patients with certain hematologic malignancies [21]. 

**Figure 1:**
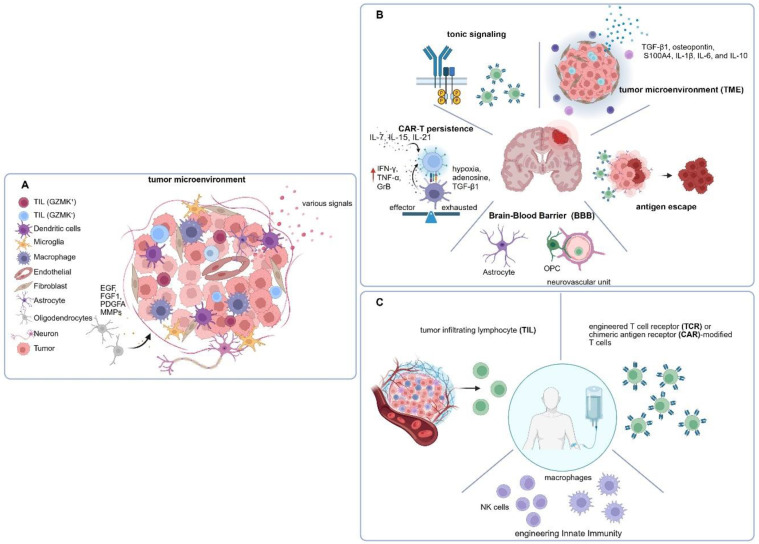
Overview of the challenges limiting chimeric antigen receptor (CAR)-based cell therapies in the suppressive glioblastoma tumor microenvironment. (**A**) The GBM TME is enriched in immunosuppressive glioma-associated myeloid cells such as macrophages and microglia, which secrete multiple inhibitory cytokines and signal mediators that limit antitumor immune responses. In addition, oligodendrocytes release cytokines and chemokines that support tumor stemness and proliferation. Bidirectional interactions between neuron and glioma cells further promote tumor growth and immunosuppression [28]. (**B**) A schematic overview illustrating key biological barriers faced by adoptive cell therapies, including tonic signaling, limited CAR-T persistence, antigen escape, immunosuppressive factors and restricted trafficking across the blood–brain barrier. (**C**) Beyond CAR-T cells, additional immune cell platforms, like tumor infiltrating lymphocytes, engineered T cell receptor or CAR-modified T cells, and CAR-engineered NKs and macrophages have also emerged as promising strategies for CAR-based cellular therapies in GBM.

**Figure 2:**
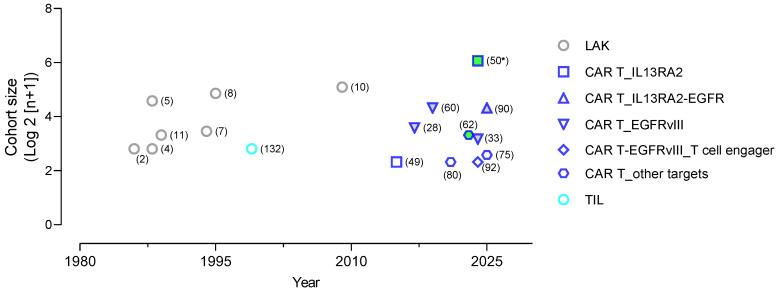
Clinical landscape of autologous T lymphocyte-based adoptive cell therapy (ACT) for adult GBM. Clinical studies published to date are summarized by publication year, cohort size (*n*, presented as log2 value of *n* + 1), therapy types (symbol shape and outline color), and outcomes (symbol fill). Open symbols (no fill) indicate studies before 2016, for which IDH mutation status is not available, or studies with *n* ≤ 5, for which outcomes were not stratified due to limited cohort size; gray fill indicates stable disease (SD) or better outcomes in <50% patients; green fill indicates SD or better outcomes in ≥50% patients; * denotes inclusion of patients with IDH-mutant tumors; numbers in parentheses, references.

The CAR-T cell approach bypasses the defective anti-cancer immune response associated with most cancer types due to immunoediting and the immune suppressive TME (Figure 1A). Therefore, CAR-T cells can be effective even when other treatments including other immunotherapies have failed. In this line, preclinical and clinical studies have investigated the application of CAR-T cells as a new therapy for diffuse glioma, especially glioblastoma, a brain cancer continuum that is poorly immune infiltrated, refractory to standard-of-care (SOC) treatment, and highly likely to re-occur after surgical resection. For GBM treatment, pioneering studies have developed CAR-T cells targeting EGFRvIII (a cancer-specific variant of EGFR), the oncogene ErbB2/HER2, and IL13RA2 (a glioma-restricted IL13 receptor) [22,23,24,25,26,27]. Preclinical studies demonstrated that these CAR-T cells exhibited anti-GBM efficacy to various extents suggesting translational promise. Subsequently, the first phase I clinical trials to explore the potential use of these CAR-T cell products in GBM patients were completed before 2017 (Figure 2) [24,27]. These studies showed favorable safety profiles of CAR-T cells administered to patients and evidence for CAR-T cell activity in clinical settings including complete tumor remission in one case [24]. However, the overall CAR-T cell response rate in glioma patients remains low and as of present, no CAR-T cell products have been approved by the FDA to move to the clinic.

Successful translation of CAR-T cell therapy through large-scale clinical trials for treating GBM has encountered multiple obstacles. Several limitations related to insufficient CAR-T cell function in solid tumor settings are not unique to glioma and GBM and have been delineated elsewhere [29,30]. Multiple mechanisms, including intrinsic signaling activity, expression density-dependent activation, and self-aggregation of CAR constructs, can induce tonic signaling, which can significantly impair CAR-T cell functionality and persistence, and increase the risk of antigen-independent or off-target activation [31,32]. Long-term exposure to antigens within the TME and undesirable CAR-T structures also pose a risk of T-cell exhaustion [29]. In addition, the blood–brain barrier (BBB) presents a unique challenge for delivery of CAR-T cell products to brain tumors (Figure 1B). Bagley et al. reported that CAR-T cells given intravenously failed to either infiltrate the glioma microenvironment or establish peripheral engraftment in de novo treatment settings without leukodepletion [33]. Although further investigations are needed to determine whether peripheral administration of CAR-T cells can infiltrate brain tumors through alternative mechanisms, such as tertiary lymphoid or lymphatic-like structures, current evidence remains limited. Consequently, most clinical trials deliver CAR-T cells via the intracranial tumor (ICT) and/or intraventricular (ICV) routes (clinicaltrials.org), which will not be discussed further. Here, we focus our discussions on targeting strategies to overcome glioma heterogeneity and treatment-induced antigen loss. 

### 2.2. CAR-T Cells Targeting Classical Tumor-Specific Antigens (TSAs)

IL13RA2 was found to be abundantly expressed in high-grade astrocytomas, but not in normal organs except for the testes [22,34]. Subsequent studies confirmed high expression of IL13RA2 protein in 38.7% of human gliomas and higher expression in most GBM tumors with wild-type IDH and *TERT* promoter mutations [35]. Despite genomic amplification, copy number gains, and *IL13RA2* mRNA being over-expressed in subsets of gliomas and GBMs, the protein function of IL13RA2 remains unclear. Due to its high affinity for IL13 and lack of a functional intracellular domain, IL13RA2 was originally considered a decoy receptor that disrupts canonical IL13 and IL4 signaling through IL13RA1 and IL4RA [36,37]. However, Bartolome et al. suggested that IL13RA2 may mediate IL13 signaling through the scaffold protein FAM120A to activate FAK and PI3K pathways in colon cancer metastasis in nude mice [38]. In addition to IL13, chitinase 3-like 1 (CHI3L1) was shown to bind to and signal via IL13RA2 and its binding partner TMEM219, mediating CHI3L1-dependent cellular and tissue responses in epithelial cells and macrophages [39,40]. In glioma cell lines, IL13RA2 was shown to bind to EGFRvIII via its cytoplasmic domain and cooperate with EGFRvIII in the activation of the downstream RAS-MAPK and STAT3 pathways [41]. Along this line, Marquez-Ortiz reported that IL13RA2 promotes proliferation and outgrowth of breast cancer brain metastases [42]. In contrast, however, Bindeman et al. demonstrated that IL13RA2 deletion promoted cell survival through increased AKT pathway signaling, and enhanced the breast-to-brain metastatic potential of the human brain-seeking breast cancer cell line MDA231BrM2 [43]. Together, these studies highlight the context-dependent and complex role of IL13RA2 via different mechanisms and in different cancer types shaped by both genetic and microenvironmental factors.

Based primarily on its tumor-restricted expression, strategies have been developed to selectively target IL13RA2. These strategies exploit site-directed IL13 mutations in residues responsible for binding to the physiological and widely expressed IL13 receptors. As such, IL13 derivatives with E13K/Y, R66D, S69D, and K105A point mutations or their combinations were vetted for enhancing the ligand-binding specificity to IL13RA2 [44]. The ligand-receptor binding affinity was demonstrated to surpass that of IL13RA2 antibodies and cognate engagement triggers internalization of the ligand-receptor complex. These findings facilitated the development of cytotoxin conjugates of the mutant ligand, which were demonstrated to have selective cell killing efficacy against IL13RA2-expressing glioma cells [44,45]. The same approach has been used for engineering CAR-T cells to target antigen-positive glioma cells [46]. Initial studies constructed first-generation CAR with the use of membrane-tethered IL13E13Y and intracellular CD3-zeta, the zetakine CAR, for IL13RA2 targeting and T cell activation, respectively [47]. Following demonstration of IL13RA2-specific anti-glioma cytolytic activity and induction of regression of orthotopic xenografts, IL13-zetakine CAR-T cells marked the first CAR-T cell clinical trial for treating recurrent GBM in a cohort of three patients showing safe and transient anti-tumor response in two patients (Figure 2) [48,49]. Newer generations of IL13 mutein CARs were subsequently engineered. The IL13BBζ version of CAR-T cells entered clinical trials and showed robust, although transient, response in one patient with multifocal recurrent GBM, leptomeningeal involvement, and spinal metastasis following progressive locoregional delivery of 16 cycles totaling ~1.4 × 10^8^ CAR-T cells [24]. A follow-up study treated a cohort of 65 patients with recurrent GBM and high-grade glioma patients [50]. Fifty percent of the patients receiving at least 3 CAR-T infusions achieved stable disease or better outcomes (Figure 2). Notably, this cohort included IDHmut tumors, which are commonly associated with longer patient survival. Although the data were not analyzed separately, 9 of the 11 of the IDHmut tumors (80%) achieved stable disease or better response and all 3 patients with complete or partial regression had IDHmut tumors [50]. Interestingly, the demonstration of CAR-T cell effectiveness in IDHmut gliomas suggests that the immune suppressive TME of IDHmut gliomas does not necessarily limit the outcome of CAR-T cell therapy.

EGFRvIII is the predominant EGFR mutation found in GBMs likely resulting from gene amplification and intragenic rearrangement leading to deletion of exon 2 through 7 in the *EGFR* gene [51,52]. Affecting a subset of approximately 30% of tumors, this cancer-specific, oncogenic EGFR variant is an attractive therapeutic target for the treatment of GBMs [53,54,55]. Multiple CAR strategies targeting EGFRvIII have been developed for preclinical and clinical studies with various strategies and extents of success [56,57,58].

Morgan et al. reported the first third-generation (CD28BBz) EGFRvIII CAR produced from a fully human monoclonal antibody, mAb 139. The engineered CAR-T cells specifically recognized and killed EGFRvIII-positive glioma stem-like cells derived from human GBMs, with no cross-reactivity to wild-type EGFR and hence no off-tumor targeting (NCT01454596) [59]. Despite the preclinical evidence of CAR activity, therapeutic efficacy was not achieved in a subsequent clinical trial of 18 patients [60]. Notably, in this study the CAR-T cells appeared to be infused intravenously, and although the presence of CAR-T cells in tumors was not determined, evidence demonstrated CAR-T cell engraftment and persistence for at least one month in peripheral blood. Additional EGFRvIII CARs engineered from 139 scFv have also been engineered and demonstrated to be able to selectively target antigen-expressing tumor cells.

Ohno et al. constructed an EGFRvIII CAR using the scFv of a monoclonal mouse antibody, 3C10, and developed 3C10 CAR-T cells (T-bodies) shown to delay xenograft tumor growth in mice [56]. A humanized scFv (2173 scFv) derived from 3C10 was developed and used to engineer CAR-T cells directed against EGFRvIII [57]. Subsequently, O’Rouke et al. reported the first CAR-T clinical trial targeting EGFRvIII in 10 recurrent GBM patients (NCT02209376) using the 2173 scFv-directed CAR-T cells (Figure 2) [27]. This study demonstrated the feasibility, safety and, notably, successful trafficking across the blood–brain barrier and infiltration into active tumor sites of the CAR-T cells following a single dose of intravenous infusion. An antigen decrease was observed in five of the seven patients receiving the CAR-T cells, suggesting on-target activity. However, the therapeutic efficacy was limited due to the continued presence of tumor cells with EGFR amplification despite loss of EGFRvIII expression. To address the antigen shift issue, Thokala et al. developed a strategy leveraging the scFv of mAb 806, a low-affinity EGFR antibody that binds EGFRvIII and other extracellular domains (ECDs) of EGFR mutants strongly, and demonstrated superior antitumor activity over the 2173 CAR-T cells in preclinical models [58].

These studies highlighted antigen escape as one of the central issues associated with current CAR-T cell approaches. The issue is particularly relevant in GBM, where tumor cells exist in heterogeneous cellular states [61]. CAR-T cell therapy-induced antigen loss is not limited to EGFRvIII-targeted therapy [62]. In addition, preclinical models are frequently designed to target constitutively expressed antigens and do not address tumor evolution in vivo. Consequently, alternative approaches are needed to overcome the current challenges, including tumor heterogeneity and antigen escape, to improve the durability of CAR-T cell therapies.

### 2.3. CAR-T Cells Targeting Unconventional Glioma Antigens

CAR-T cell studies as discussed above focused primarily on GBM-specific CAR targets, for on-target off tumor effects are considered the least desirable. This strategy of targeting classical TSAs, however, has suffered from intrinsic and induced resistance due to expression heterogeneity and on-target editing through multiple mechanisms with CAR-induced trogocytosis of cognate antigen contributing to not only antigen loss but also CAR-T cell dysfunction [63,64]. None of the most common CAR targets selected in previous studies showed uniform expression on all or most GBM cells. Although target expression can be determined prior to CAR-T cell administration, tumor heterogeneity and technical challenges may prevent reliable assessment of the prevalence of antigen-positive cancer cells [65]. In addition, the underlying mechanisms of CAR-T cell targeting are not fully understood as EGFRvIII appears to be a moving target even in the absence of on-target selection pressure and CAR-T cell-induced editing [63]. Thus, new antigen-vetting tactics are needed to increase cancer cell coverage and targeting efficiency.

One such strategy is the GD2 CAR that targets disialoganglioside GD2, a sialic acid-containing glycosphingolipid expressed primarily on the cell surface. Unlike other gangliosides that are widely found in normal tissues, GD2 was among a small number of vetted targetable tumor antigens with relative specificity for some tumors including neuroblastoma and GBM [66,67,68]. GD2 CAR-T cells were developed for the treatment of GBM and other brain tumors and tested in clinical trials safely [62]. Despite the presence of GD2 in the normal peripheral and central nervous systems and GD2 antibody therapy being associated with neurological side effects [67,69], GD2 CAR-T cells administered directly to CNS tumors appeared to be well-tolerated and remarkably efficacious in treating midline pediatric gliomas [70]. Other preclinical work also showed better tumor control by enhancing GD2 CAR-T cells with IL-15 transgenes, leading to more persistent CAR-T cells without exceeding dose limits [71].

Unlike GD2, chlorotoxin is a 36-amino acid peptide found in the venom of the deathstalker scorpion (*Leiurus quinquestriatus*), which was originally used to block small conductance chloride channels [72]. However, chlorotoxin injected per se is not toxic to humans, which suggests its lack of major impact on normal tissues in physiological settings. Ullrich et al. described a glioma-specific chlorotoxin-sensitive chloride current, and subsequent studies further showed that chlorotoxin exhibited a glioma-specific affinity and the ability to cross the BBB in glioma patients leading to its use as a natural agent for diagnosis and tumor targeting [72,73]. Wang et al. developed an unconventional approach using chlorotoxin to redirect cytotoxic T cells, CLTX CAR-T cells, to target GBM in a preclinical study [74]. They showed that CLTX CAR-T cells are safe in mice and effective in mediating anti-cancer activity in vitro and in mice bearing established GBM xenografts. A subsequent clinical trial (NCT04214392) demonstrated the feasibility, safety, and notable efficacy in GBM patients (Figure 2) [75]. Importantly, CLTX CAR-T cells did not induce antigen loss as seen with both EGFRvIII and IL13RA2 CAR-T cells [24,27,63]. Chlorotoxin has multiple putative glioma-specific cell surface receptors including MMP2, CLCN3, and Annexin A2, whereas only MMP2 appeared to be required for CLTX CAR-T cell activation [74]. It is currently unclear whether such specificity is conferred by the specific CAR structure and/or relative antigen density, and it may be worth testing whether scFv can be used to target MMP2, CLCN3, and Annexin 2 individually or in combination. Future studies are warranted to investigate the mechanisms underpinning CLTX CAR-target interaction that might be fine-tuned to improve CAR-T cell function.

### 2.4. Emerging CAR-Directed Targeting

Although pan-cancer essential genes are generally considered less favorable targets for conventional drug development [76], the most successful CAR-T cells are those targeting CD19, which is shared by a wide spectrum of hematological malignancies. CAR-T cell therapy is not necessarily dependent on inhibiting downstream mechanisms of the target antigen. CAR-T cells have been developed to target tumor-associated antigens such as EphA2, B7-H3, CD70, IL1RAP, CD99, and the STEAP family metalloreductases, which are not cancer lineage-specific but are expressed in GBMs [77,78,79,80]. CAR-T cells redirected to some of these targets have entered clinical trials for GBM treatment (e.g., NCT05366179). However, some of these antigens are also expressed on T cells, raising concerns about potential on-target, off-tumor effects, including auto-targeting and fratricide among CAR and non-CAR T cells, and requiring innovative targeting strategies [77,81,82,83]. In addition, *PTPRZ1* has been identified as an antigen-prolific gene highly expressed in majority of gliomas, giving rise to multiple recurrent tumor antigens in tumor vaccine discovery studies. Accordingly, PTPRZ1-targeting immunotherapies including vaccine-based and T cell-based approaches have been developed for GBM treatment [84,85,86,87]. However, *PTPRZ1* is also abundantly expressed in normal brain tissue. Although genetic deletion of Ptprz1 in mice resulted in only mild neurological defects [88], the clinical safety of CAR-T cells targeting bulk PTPRZ1 expression, rather than HLA-presented tumor-derived peptide epitopes, remains unclear, particularly given the risk of on-target, off-tumor neurotoxicity. 

### 2.5. Combinatory Targeting

Intuitively, the extensive intra-tumoral heterogeneity of GBMs that has tremendously impeded progress in applications of CAR-T cells for GBM management can be mitigated by using multivalent CARs to increase target coverage. To this end, CAR-T cells redirected to two or more of the common glioma targets, EGFR, EGFRvIII, IL13RA2, HER2, and EphA2, have been developed. This was achieved through diverse engineering strategies including tandemization of multiple antigen-binding domains [89], dualization by co-expression of two different CARs [90], combination with T cell engagers [91,92], and leveraging scFv versatility [58,93,94,95]. Interestingly, quadrivalent CAR-T cell products targeting B7-H3, EGFR806, HER2, and IL13-zetakine are being explored in an ongoing clinical trial for children and young adults with diffuse midline glioma (NCT05768880), although similar aggressive approaches have not been reported for GBMs.

Notably, Roybal et al. developed a SynNotch CAR strategy, where a prime-and-kill genetic circuit was devised to drive local CAR expression and achieve multivalent CARs [96,97,98]. This approach allows imperfect glioma antigens such as EphA2 and IL13RA2, which are also expressed in normal tissue outside the CNS, to be targeted by CAR-T cells without on-target, off tumor side effects. In addition, synNotch CAR-T cells were shown to be less susceptible to exhaustion and preclinical studies demonstrated improved anti-tumor activity as compared to conventional CAR-T cells [97,98,99]. Currently, a phase 1 clinical trial is testing the eSYNC CAR-T cells where an anti-EGFRvIII synNotch receptor is used to induce anti-EphA2/IL13RA2 CAR expression in patients with EGFRvIII^+^ GBMs (NCT06186401). 

Future studies may consider combining these complementary strategies, especially, next-generation designs of SynNotch CAR-T cells targeting chlorotoxin receptors, B7H3, CD70, CD99, and other emerging candidates to increase CAR specificity and limit neurotoxicity. In parallel, new target discovery and rational antigen pairing can further benefit from analyzing and incorporating data obtained from scRNAseq studies and other currently available technologies. Collectively, these approaches may hold promises to overcome the therapy hurdles imposed by glioma heterogeneity and the immune suppressive TME, ultimately leading to improved clinical translation of CAR-T cell therapies for GBM.

## 3. CAR-NK Cell Therapy

NK cells are a specialized subset of innate lymphoid cells that play a central role in immune surveillance. NK cells constitute nearly 5–20% of circulating lymphocytes in humans and act as early responders against diverse pathogen challenges and early signs of cancer. Unlike classical T lymphocytes, NK cells recognize heterogenous tumor populations through a broad repertoire of activating and inhibitory receptors, even in the absence or downregulation of major histocompatibility complex class I (MHC-I). Importantly, NK cells rarely induce graft-versus-host disease (GVHD), even in allogenic “off-the-shelf” applications [100,101]. In addition, NK cells secrete cytokines such as IFNγ and TNFα, which play critical roles in immunomodulation and shaping the TME [102,103]. Collectively, these functions facilitate the activation of cancer immunity cycle, offering a therapeutic strategy to counteract the immunosuppressive microenvironment of GBM. The therapeutic utility of CAR-engineered NK cells (Figure 1C) for the treatment of GBM has so far mainly been investigated in preclinical studies.

Muller et al. generated CAR-NK cells using the scFv of an EGFRvIII-specific antibody fused to an intracellular DNAX-activation protein 12 (DAP12) signaling domain and the chemokine receptor CXCR4 was co-expressed to enhance migration toward tumor site. Intravenous administration into mice bearing EGFRvIII-positive GBM cells significantly inhibited tumor growth and extended survival [104]. To prevent immune escape mediated by wild-type EGFR clones, Genssler et al. engineered dual-targeting CAR-NK cells recognizing both EGFR and EGFRvIII and showed that this strategy outperformed monospecific CAR-NK cells in targeting established glioblastoma cell lines [105].

Chondroitin sulfate proteoglycan 4 (CSPG4, a.k.a., NG2 for neuroglial antigen 2) has emerged as another promising CAR target. CSPG4 is highly expressed in ~50% of GBMs and is associated with poor prognosis [106,107]. Functionally, CSPG4 contributes to malignant progression by promoting angiogenesis and mediating the interaction between tumor cells and collagen [108,109]. Building on this functional characterization, CAR-NK cells were generated by incorporating the CSPG4-specific scFv 763.74 fused with CD28-CD3ζ activation domains and demonstrated to have robust specificity against CSPG4-positive U87 cells [106].

B7-H3 has recently emerged as a promising GBM target with elevated expression correlating with higher glioma grades and certain GBM cell lines [110]. Chaudhry et al. showed that NK cells co-expressing a B7H3 CAR and a TGF-β1 dominant negative receptor (DNR) retained cytotoxicity even in the presence of TGF-β1 [111], highlighting a strategy to circumvent tumor-mediated immunosuppression.

Beyond autologous NK cells, NK-92-derived CAR-NK cells targeting HER2 via an FRP5 scFv-CD28-CD3ζ construct demonstrated selective cytotoxicity against HER2-positive xenograft tumors [112,113].

## 4. CAR-Macrophages (CAR-MACs)

Glioma-associated myeloid cells, often referred to as glioma-associated macrophages (GAMs), comprising both microglia (embryonic origin) and monocyte-derived macrophages (bone marrow origin), which act as key immunomodulators within the TME. These populations contribute to immune suppression through multiple mechanisms, including expression of immune checkpoint ligands (e.g., PD-L1), secretion of anti-inflammatory cytokines such as IL-10 and TGF-β, metabolic disruption via arginase-1 activity, and impaired antigen presentation associated with low HLA-DR expression [114,115].

While these cells were historically categorized into M1 and M2 phenotypes, recent single-cell RNA sequencing studies reveal a continuum of activation states. Notably, emerging data describe early and mature myeloid-derived suppressor cell-like populations (E-MDSC and M-MDSC) [116], suggesting transitional states along the monocyte-macrophage lineage.

Macrophages (MACs) are the most abundant infiltrating immune cell population within the brain TME, which play a critical role in innate immunity [117]. Their high infiltration capacity, low toxicity, and cytotoxicity potential against tumor cells make them attractive candidates for cellular immunotherapy, an area dominated by approaches leveraging adaptive immune cells [118].

Klichinsky et al. generated CAR-MACs by transducing the THP1 cell line with a first-generation CD19 CAR construct containing the CD3ζ intracellular signaling domain. CD3ζ is homologous to the Fc common γ-chain, FcεRIγ, which can mediate antibody-dependent cellular phagocytosis (ADCP) in macrophages [119]. In this study, CAR-MACs were shown to infiltrate the TME, mediate antigen-specific phagocytosis of target tumor cells, and boost anti-tumor T cell activity. These findings provided proof-of-concept that prompted the preclinical development of multiple CAR-MACs targeting various glioma-associated antigens, such as EGFRvIII, CD133, and HER2 [120,121]. Further, Lei et al. developed a second-generation CAR by including the TLR4 signaling domain in addition to CD3ζ and showed strong antitumor activity of this CAR targeting EGFRvIII+ U87 cells [122].

In addition, tumor-associated macrophages (TAMs) are known to drive immunosuppression in cancer. One of the SIGLEC family members, SIGLEC9, was found to be highly expressed in monocyte-derived TAM populations enriched in GBMs that do not respond to immune checkpoint inhibitors [123]. Targeting murine Siglec-9 was shown to synergize with anti-PD1/PD-L1 treatment. Fu et al. reported an in situ synthetic SIGLEC9-based chimeric switch receptors (CSRs) designed to convert inhibitory to activating signaling by replacing the intracellular domain of SIGLEC9 with that of TLR4 and CD40. This approach was demonstrated to elicit innate and adaptive antitumor immunity and promote CAR-mediated targeting of IL13RA2+ murine glioma cell lines CT2A and GL261 [124].

## 5. Glioma TIL and TIL Therapies

CAR-T cells are designed to target predefined tumor antigens through ex vivo engineering, and although multivalent CAR-T cells are possible, target selection remains intrinsically limited by antigen availability, specificity, and heterogeneity. Unlike CAR-T cells, tumor-infiltrating lymphocytes (TILs) are naturally occurring, tumor-reactive T cells that exert anti-cancer T cells without genetic engineering and manipulation. In principle, TILs possess broad clonal diversity capable of recognizing heterogeneous tumor antigens including “unknown”, cryptic, and unconventional neoantigens that elude common antigen discovery and targeting strategies [85]. Moreover, TILs derived from solid tumors are expected to be tumor penetrant, given their established routes of tumor infiltration and residence within the TME [125]. 

Consistent with these attributes, TIL therapy has demonstrated clinical efficacy in patients with advanced and immune checkpoint inhibitor-resistant human cancers [126,127,128,129,130,131], culminating in FDA approval of lifileucel, a one-time TIL therapy for advanced melanoma. Despite this progress, TIL therapy in gliomas remains understudied. The only clinical study, published in 1999 by Quattrocchi et al., demonstrated feasibility and preliminary efficacy (Figure 2) [132]. This work, however, was followed by a prolonged hiatus, with a very limited number of clinical trials registered as of 2025 (NCT03347097; NCT06640582), both of which are combined with immune checkpoint blockade. The slow advancement likely reflects several challenges, including the immune-suppressive TME, the scarcity of TILs, difficulties in ex vivo expansion, and issues related to phenotypic drift accompanied by loss of cytotoxic function [133,134].

GBM TILs are primarily granzyme K-positive (GZMK+) effector T cells, a phenotype also observed in TILs from other solid tumors. Although the biological role of the GZMK+ phenotype has not been fully defined, recent studies demonstrated that effector T cells constitutively secrete GZMK, which can cleave and activate multiple members of the complement cascade, including C3 and is sufficient to trigger full complement activation [135,136]. Notably, GBM cells, particularly those in the mesenchymal state, express multiple complement components [137]. In addition, the glioma TME is enriched in microglia and other myeloid cells, which can also supply precursor complement molecules [138].

These observations suggest that, beyond its canonical role in cytotoxicity in concert with perforin, GZMK may function as an immunomodulatory mediator, interfacing with tumor cells and non-tumor stromal and immune cell populations to shape the evolving immune TME. Consistent with this model, the mesenchymal state of GBM is typically associated with a T cell-inflamed phenotype, despite GBMs being broadly classified as an immunologically cold tumor type. Unfortunately, this effector TIL population central to antitumor immunity particularly in the GBM TME cannot be stably maintained during ex vivo expansion of isolated GBM TILs [134]. The mechanisms underlying this phenotypic shift following removal from the native TME remain poorly understood. However, multiple factors are likely contributory, including loss of tumor antigen exposure, absence of antigen-presenting and other supporting cells, disruption of metabolic programing, and alterations in epigenetic and cytokine signaling, particularly the reliance on nonphysiological expansion protocols.

## 6. Conclusions

Although immunotherapies targeting major immune checkpoints have provided durable benefits in several malignancies, they have thus far benefited only a small fraction of patients with GBM [139,140]. Consequently, adoptive cell therapies have been actively pursued as an alternative approach with the potential to benefit a broader patient population, as summarized in Figure 2.

CAR-T cells have achieved unprecedented success in the treatment of hematological malignancies. However, CD19 CAR-T cells are intrinsically well matched to the hematologic context, and truly CD19-like targets—tightly lineage-restricted, uniformly expressed, and dispensable to normal tissues—are uncommon in solid tumors, likely numbering only a few dozen at most [141,142].

Experience from early clinical studies in GBMs, particularly those evaluating CAR-T cell therapies, has highlighted two recurrent challenges [30,118,143]. First, antigen escape is a frequent mechanism of treatment failure and is unlikely to be fully overcome by targeting single antigens or through limited combinatorial strategies. Second, poor persistence and functional durability of transferred T cells remain major obstacles, suggesting inadequate fitness and maladaptation of peripheral T cells within the highly suppressive GBM TME, even after extensive genetic engineering.

Recent advances in tumor immunology have provided deeper insights into the GBM TME, including the mechanisms governing the coevolution of tumor-infiltrating lymphocytes (TILs) with malignant and non-malignant stromal and immune cells. In contrast to engineered T cell products, TILs possess greater resilience to the TME and inherent clonal diversity, which may render them less susceptible to antigen escape and provide a biologically distinct therapeutic strategy. Indeed, the recent clinical success of lifileucel and TCR-engineered autoleucel in solid tumors signals a shift toward biology-constrained engineering strategies that more closely align with physiological T-cell recognition and signaling [144,145]. We propose that tumor microenvironment–tuned TIL expansion approaches adapted to physiological TIL biology and the epigenetic, metabolic, and cytokine milieu supporting TILs may enhance manufacturing feasibility while improving T cell fitness, antitumor efficacy, and long-term persistence.

## Data Availability

No new data were created or analyzed in this study.
